# Bacterial toxins: Offensive, defensive, or something else altogether?

**DOI:** 10.1371/journal.ppat.1006452

**Published:** 2017-09-21

**Authors:** Justine K. Rudkin, Rachel M. McLoughlin, Andrew Preston, Ruth C. Massey

**Affiliations:** 1 School of Microbiology, University College Cork, Cork, Ireland; 2 APC Microbiome Institute, University College Cork, Cork, Ireland; 3 Host-Pathogen Interactions Group, School of Biochemistry and Immunology, Trinity Biomedical Sciences Institute, Trinity College Dublin, Dublin, Ireland; 4 Department of Biology and Biochemistry and the Milner Centre for Evolution, University of Bath, United Kingdom; 5 School of Cellular and Molecular Medicine, University of Bristol, Bristol, United Kingdom; Stony Brook University, UNITED STATES

## Abstract

The secretion of proteins that damage host tissue is well established as integral to the infectious processes of many bacterial pathogens. However, recent advances in our understanding of the activity of toxins suggest that the attributes we have assigned to them from early *in vitro* experimentation have misled us into thinking of them as merely destructive tools. Here, we will discuss the multifarious ways in which toxins contribute to the lifestyle of bacteria and, by considering their activity from an evolutionary perspective, demonstrate how this extends far beyond their ability to destroy host tissue.

## Background

In the century since the existence of bacterial toxins was first conceived, we have learned many intricate details of their regulation, secretion, 3D structures, target receptors, and mode of action. Their undisputed offensive role in causing the tissue damage associated with many infectious diseases has understandably led us to view them from a disease-centric perspective. However, if we take a step back and look beyond an individual patient, the selective advantage that some toxins confer to the producing bacteria becomes unclear. While, for many bacteria, there is a tangible benefit to producing toxins in that they directly contribute to their replication and transmission to new hosts [1, 2], there are several for which it is not clear how causing disease symptoms is of any selective advantage to the bacteria. In some cases, it can even seem disadvantageous to produce toxins, as the resulting pathology results in an evolutionary dead end for the pathogen [2].

To understand this apparent paradox, we need to consider the many levels at which selection works on pathogens. Our early musings on the evolution of virulence led many to believe that microbial pathogens should evolve towards a benign coexistence with their host to avoid limiting their own replication through either the death or isolation of the host. As we have learned more about how microbes transmit between hosts and about the competition that exists between microbes within a host, we have come to understand that the evolution of virulence is considerably more complex than we originally appreciated. While a disease-centric viewpoint will help us understand the immediate consequences of toxin expression, we need to look more broadly if we are to fully understand toxins. As there are many excellent reviews describing the role toxins play in causing tissue damage and disease symptoms [3–5], we will instead focus on examples of bacterial toxins for which the contribution to the long-term existence and survival of the bacteria has been unclear until recently. By examining the less offensive, non-tissue-destructive activities of bacterial toxins, we will discuss their more subtle roles in subverting host immunity (defensive) and will also discuss some recent findings that suggest toxins can act in neither an offensive nor defensive role but instead provide benefits to the bacteria unrelated to a direct interaction with their host, such as facilitating biofilm formation, motility, and niche establishment.

## Adenylate cyclase–affecting toxins: A role beyond pathogen transmission

The classic example of a bacterial toxin that affects the adenylate cyclase (AC) activity of its host is cholera toxin. However, many diverse genera of bacteria express similarly acting toxins, including other enteropathogens such as *Escherichia coli* and *Clostridium perfringens* but also respiratory pathogens such as *Bordetella pertussis*. For the enteropathogens, the link between the offensive activity of these toxins and the selective advantage they confer is clear; by interfering with the AC system of the cell they attach to, they activate the cell’s calcium channels, leading to a release of ions from the cell into the lumen of the gut, causing the subsequent release of water to balance out ion-induced osmotic stress [1]. This results in the production of diarrhoea and the subsequent transmission and ongoing survival of the bacteria [1].

It is interesting to consider the role such a toxin would play for a respiratory pathogen. *B*. *pertussis*, the causative agent of pertussis (commonly referred to as whooping cough), produces 2 well-characterised toxins, pertussis toxin (PT) and the adenylate cyclase toxin (ACT). ACT has direct cyclase activity, whereas PT is an adenosine diphosphate (ADP)-ribosyltransferase that modifies the alpha subunit of heterotrimeric guanine nucleotide-binding (G) proteins of host cells [5]. A consequence of the aberrant signalling arising from this can be uncontrolled activation of host cell AC [5]. Thus, both of these toxins can cause hugely elevated levels of cAMP within host cells.

**Fig 1:**
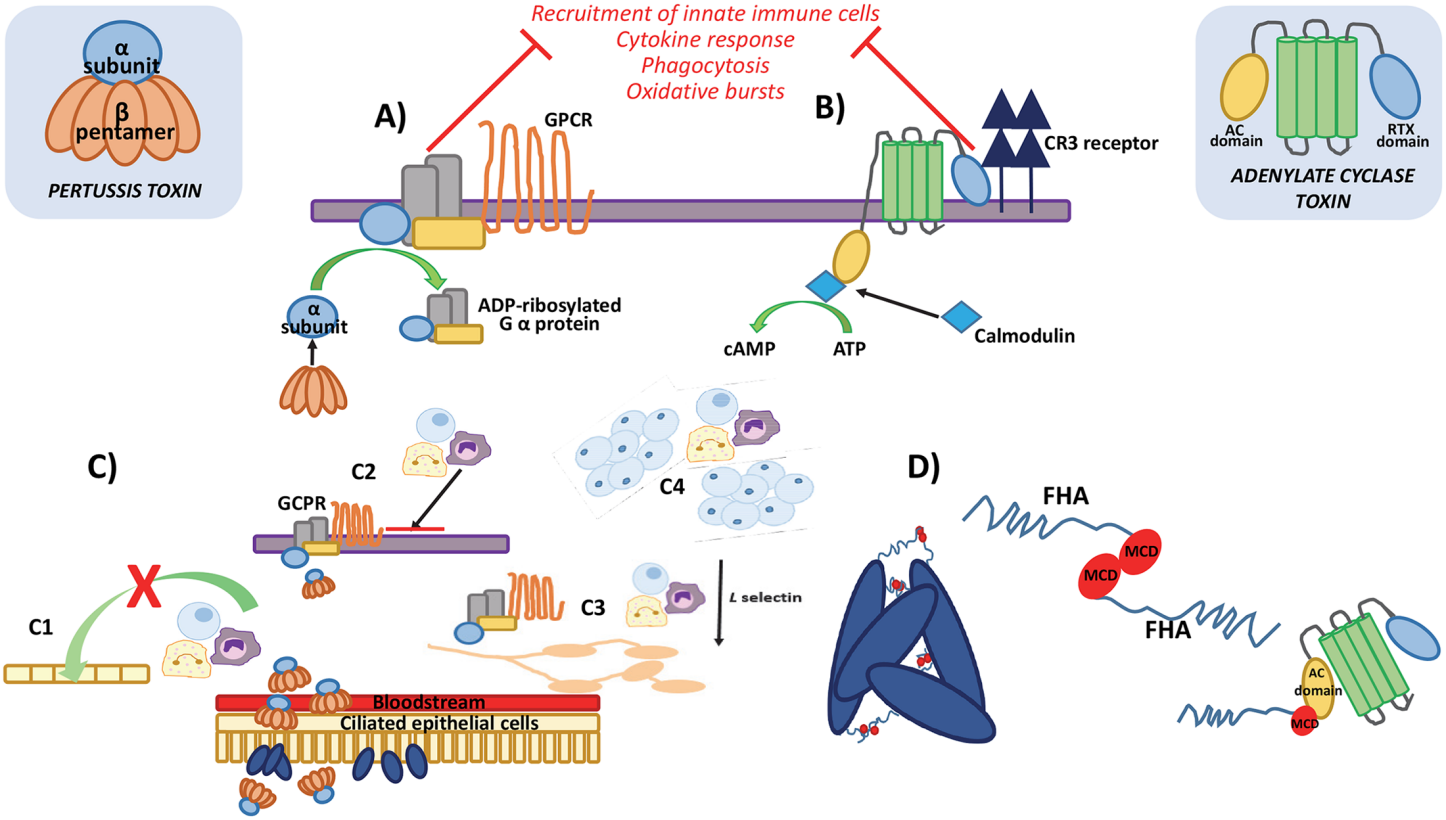
Contribution of pertussis toxin (PT) and adenylate cyclase toxin (ACT) to pathogenicity of *Bordetella pertussis*. The adenylate cyclase (AC)-affecting toxins of *B*. *pertussis* contribute to disease progression via: **(A)** PT is endocytosed into a cell and, following intracellular processing by the endoplasmic reticulum, the alpha subunit is released into the cytosol. This subunit ADP-ribosylates the alpha subunit of G proteins, disassociating it from its G protein coupled receptor (GPCR) on the cell surface inhibiting recruitment of immune cells to the site of infection. **(B)** ACT interacts with cell surface complement receptor (CR3) on macrophages and neutrophils, affecting antigen presentation and recruitment of the downstream adaptive immune response. The AC domain translocates to the cell cytoplasm and is stimulated upon calmodulin binding, leading to increased cAMP levels, inhibiting pro-inflammatory cytokine release and complementing mediated phagocytosis, and interfering with immune cell recruitment. **(C)** PT released into the bloodstream from cells growing on ciliated epithelial lung cells has been shown to contribute to development of leukocytosis. The mechanism is unclear but several have been proposed including **(C1)** PT inhibiting migration of lymphocytes across epithelium layers, **(C2)** PT interfering with GPCR signalling, effecting immune cell recruitment, **(C3)** PT inhibiting GPCRs required for leukocytes to stick to lymph nodes, interfering with extravasation, and **(C4)** PT stimulating the expansion of normal naïve immune cells and not proliferation of activated cells. **(D)** ACT inhibits biofilm formation by interfering with filamentous haemagglutinin–filamentous haemagglutinin (FHA-FHA) interactions between cells. The AC domain of the toxin binds to the mature C-terminal domain (MCD) at the distal tip of the FHA protein, blocking its function in biofilm.

At a superficial level, it might seem reasonable to suppose that toxin-induced secretion of ions and water from the cells lining the lungs would cause the host to cough and expel the bacteria, resulting in its onwards transmission. However, the availability of appropriate animal models limits our understanding of the role the distinctive cough plays, as mice do not cough in response to any stimulus. As such, the contribution the cough makes to disease progression and onwards transmission of these bacteria is unproven. What we do know is that comparisons between wild-type and PT-deficient strains have identified a role for PT in modulating host immune responses [6–8]. Despite the moniker, the most definite effect of pertussis in infants is leukocytosis, and PT is believed to directly contribute to this [9]. High levels of leukocytosis is associated with severe pertussis and attributed to causing pulmonary hypertension, leading to cardiac failure—the main cause of pertussis-related death in infants [10]. In apparent contradiction to this, PT has been shown to inhibit chemokine production by cells in the lung shortly after initial inoculation, which reduces the recruitment of neutrophils to the site of infection [11–14]. The antibacterial functions of resident airway macrophages are also inhibited by PT, although the specific signalling mechanisms behind these are unclear [15]. These defensive effects suppress the host’s control of *B*. *pertussis* growth early in infection, aiding the establishment and development of the infection [6,14]. However, these suppressive effects appear to switch at later time points when PT production appears responsible for pro-inflammatory effects, either through promoting inflammation per se or by inhibiting its resolution [16]. Thus, PT appears to have direct pathological effects such as stimulation of leukocytosis, as well as defensive properties through modulation of immune functions, suggesting equally defensive and offensive roles for this toxin (summarised in Fig 1).

For ACT, the resulting rapid increase in cellular cAMP as a result of its AC activity inhibits a number of antibacterial activities, including phagocytosis of the bacteria, induction of the oxidative burst in neutrophils, and inhibition of reactive oxygen species production [17–21]. The inhibition of these activities suppresses innate immunity control of *B*. *pertussis* during early infection [22,23]. Furthermore, it is thought that targeting of complement receptor (CR3)-expressing dendritic cells affects antigen processing by these cells and, in doing so, affects the ensuing adaptive immune response to infection [24]. Thus, ACT has key defensive activities during infection. Interestingly, however, ACT has also recently been shown to affect the ability of *B*. *pertussis* to form biofilms [25]. While primarily studied in vitro, it is hypothesised that *B*. *pertussis* biofilms are important for growth and persistence in the nasopharynx during infection. The key adhesin, filamentous haemagglutinin (FHA), is heavily involved in *B*. *pertussis* biofilm formation and development [26]. Interestingly, the AC domain of ACT can bind to FHA and, in doing so, inhibit biofilm formation. Binding is through the catalytic domain of AC but is independent of catalytic function. Exogenous AC can also disrupt preformed biofilms [25]. Expression of FHA and AC is regulated by the activity of the *Bordetella* virulence gene (Bvg) 2-component system [27,28]. Differential expression of FHA and AC could alter the balance between biofilm formation, nonbiofilm growth, and possibly dispersal of *B*. *pertussis* from established biofilms. Thus, AC could have an important role in regulating the mode of growth of *B*. *pertussis* during infection, in addition to its multiple roles in modifying host responses (summarised in Fig 1).

Therefore, it appears that the contribution of AC-affecting toxins to the life cycle of pathogenic bacteria may be considerably more complex and involve behaviours far and beyond the offensive, playing defensive immune modulation functions, altering the mode of growth, and aiding in niche establishment and bacterial dispersal to new sites of colonisation or infection.

## Host cell membrane destruction: Asymptomatic carriage and niche establishment

The haemolytic capability of bacteria was one of the first true virulence factors identified for bacteria, and the major mechanism through which this occurs is the formation of pores in the host cell membranes, causing them to lyse. Several genera of bacteria utilise this type of toxin, including *Listeria monocytogenes* [29], *Streptococcal* species [30–33], *Salmonella* species [34, 35], and *E*. *coli* [36–38]. However, it is *Staphylococcus aureus* that appears to make the most use of this type of toxin, in which up to 7 distinct multicomponent pore-forming toxins have been identified (alpha, gamma, PVL, LukAB, LukED, and LukMF) [39]. The undeniable offensive capabilities of these types of toxins and the role they play in the development of infections is clear. What is less clear is why, given that as many as 60% of us can carry this bacterium in our noses asymptomatically, does it maintain such potentially pathogenic capabilities?

To understand this, we need to consider the 3 distinct ways in which we interact with this bacterium: asymptomatic carriage, superficial skin and soft tissue infections (SSTIs), and invasive disease (e.g., bacteraemia, pneumonia, etc.). Understandably, much of the focus on these toxins has been on how they contribute to interactions that result in the most severe types of infection caused by this bacterium, invasive disease. Animal models clearly demonstrate the destructive contribution these toxins make to the development and severity of invasive diseases (an excellent summary table of such studies is provided in [39]). However, from an evolutionary perspective, as the bacteria rarely transmit from an invasive infection to another person, these infections represent a dead end for the bacteria, and so the selective advantage the toxins confer to the bacteria during invasive disease is, if anything, negative [2].

Consideration of SSTIs does provide some explanation for the long-term benefit of producing toxins; however, it requires us to merge our appreciation of offensive and defensive activities; if the cell types killed by the toxins are the cellular components of host immunity, then they can be simultaneously offensive and defensive. In addition to killing leukocytes, which enables the bacteria to survive the onslaught of the immune system, *S*. *aureus* SSTIs are notorious for the amount of purulent material that is produced, and this feature has been shown in many studies to be directly affected by toxins [40–42]. The major components of pus are bacteria and dead neutrophils, which, with its physically sticky nature, means it is a very effective means of transmission for *S*. *aureus* [43]. Thus, there is a clear advantage to the production of toxins during SSTIs.

Ultimately, however, we need to consider the role these toxins play in what is by far its most common niche, the nose, as invasive disease and SSTIs represents only a fraction of the interactions that occur between us and this bacterium. In reality, the human immune system is about 1,000-fold more likely to encounter *S*. *aureus* in the context of colonisation than it is to encounter it during a pathogenic infection [44]. Whilst it’s tempting to think that the lysis of immune cells might facilitate the ability of *S*. *aureus* to colonise the nose, the exogeneous destruction of cells and tissue would result in the triggering of inflammatory processes, which is neither a feature associated with carriage of *S*. *aureus* nor conducive to long-term colonisation of this niche. In a recent population-based study, we sought to compare the toxicity of isolates from healthy noses to those from invasive diseases. As toxin production is readily switched off by spontaneous mutations in toxin-regulating loci such as the accessory gene regulator (*agr*) and the regulator of surface proteins (*rsp*), were toxins not playing an important role during carriage, one might expect to see many of these mutants arising in the nose. However, we found the opposite in that the carriage strains were significantly more toxic than the invasive strains [2, 45], suggesting that there is strong selection for toxin expression in this niche.

**Fig 2:**
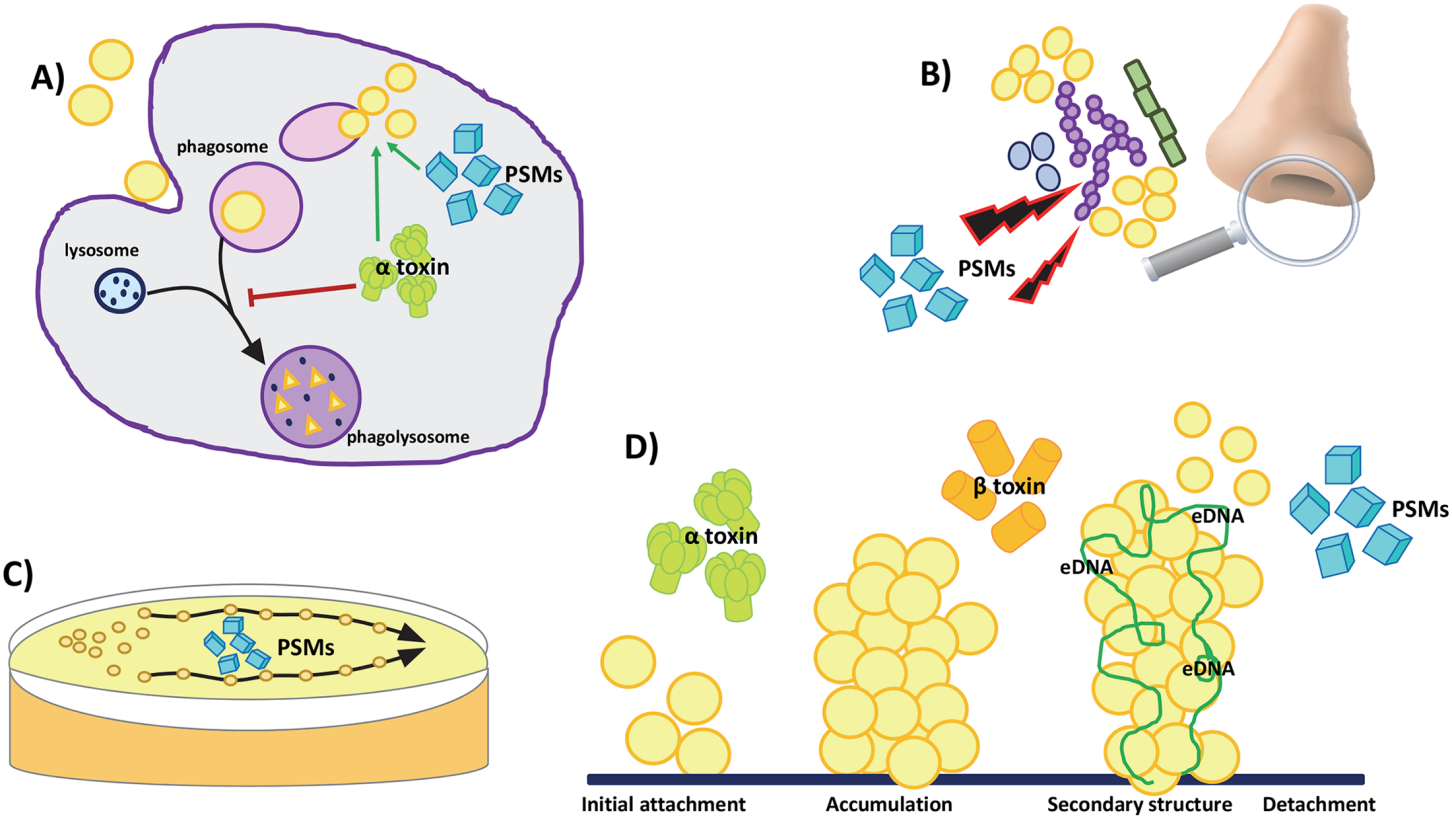
The host cell membrane attacking toxins of *Staphylococcus aureus* and their roles beyond host cell lysis. **(A)** Phagocytosis of invading bacteria is followed by fusing of the phagosome to the lysosome, resulting in destruction of the bacteria. *S*. *aureus* alpha (α) and phenol-soluble modulin (PSM) toxins inhibit fusing of the lysosome. This enables the bacteria to escape from the phagosome into the cytoplasm, allowing intracellular niche establishment and replication. **(B)** PSM toxins target cohabiting bacterial species within established niches, aiding in competition for resources and competitive exclusion of nonkin isolates. **(C)** PSM toxins have surfactant properties in vitro, enabling sliding movement across agar surfaces in the absence of traditional mobility structures such as flagella and pili. **(D)** Pore-forming toxins are involved at each step of *S*. *aureus* biofilm formation. During the initial cell attachment phase, alpha-toxin is involved in establishing cell-to-cell contacts, enabling the formation of secondary biofilm structures. In the later stages of the biofilm lifestyle, extracellular matrices develop, surrounding the cells within the biofilm. In the presence of extracellular DNA (eDNA), beta-toxin covalently cross-links with itself, adding to this extracellular nucleoprotein biofilm matrix and contributing to the formation of complex biofilm secondary structuring. Detachment from the mature biofilm allows for dispersal to new sites of infection. PSM toxins are involved in this stage of the biofilm lifestyle, aiding release of cell clusters from the main body of the biofilm.

We therefore need to consider what else these toxins are doing in the nose (summarised in Fig 2). There is conflicting evidence on whether *S*. *aureus* commonly forms biofilm in the human nose or lives in a more dispersed manner. With a recent study finding that 60% of chronic rhinosinusitis patients have evidence of noninvasive *S*. *aureus* biofilm in their noses [46], it is worth speculating about whether toxins contribute to this. The development of a biofilm involves initial attachment to a surface and accumulation of an extracellular matrix, which is largely comprised of cell surface polysaccharides, extracellular DNA (eDNA), and proteins. During the initial attachment stages, the *S*. *aureus* beta toxin (a sphingomyelinase) has been shown to play a role in the production of an insoluble extracellular nucleoprotein matrix surrounding the cells in the biofilm matrix in vitro. Secreted beta toxin covalently cross-links with itself in the presence of DNA to form oligomers, which promote biofilm formation, with beta toxin mutants not adhering as well as their isogenic counterparts in vitro [47]. Also, beta toxin has been implicated in biofilm formation in vivo during endocarditis infections, with reduced vegetation mass formed by isogenic beta-toxin mutants compared to beta toxin–positive wild types [47]. In addition, alpha toxin has been shown to play a role in initial cell-to-cell contacts within the biofilm. Whilst alpha toxin mutants are able to colonise a surface, they don’t organise into multicellular macrocolonies and lack secondary biofilm structure, indicating a role for this toxin in the middle stages of biofilm development [48]. It is therefore possible that the selective advantage these pore-forming toxins confer is to enhance colonisation of the nose via their effects on biofilm formation.

In addition to their potential role in the formation of biofilm, we believe it is possible that pore-forming toxins also enhance the ability of the bacteria to colonise the nose by manipulating rather than killing host immune cells. Recently, we have shown that during the establishment of *S*. *aureus* nasal colonisation in experimental systems, there is an accumulation of phagocytes (both neutrophils and macrophages) within the nasal tissue [49]. The coexistence of these cell types (bacteria and phagocyte), each with the potential to kill the other, suggests they are existing in some sort of homeostasis, and when the following studies are considered, it is possible that the toxins are instead manipulating these immune cells to facilitate their coexistence. There are several recent papers that show that, once taken up by phagocytes, *S*. *aureus* pore-forming toxins such as alpha toxin facilitate escape from the phagosome, enabling the bacteria to enter the cytoplasm and replicate and establish an intracellular niche [50]. A role for such toxins has been found also in the subversion of normal autophagic processes. Autophagy is an important homeostatic process in eukaryotic cells in which damaged cytosolic components are removed and recycled in double-membrane vacuoles called autophagosomes, which fuse with lysosomes and are digested [51]. Autophagy plays an important role in the host’s defence against invasive or intracellular pathogens [52–54]. *S*. *aureus*’s ability to subvert autophagy is under the control of the major regulator of toxin expression, the Agr system, and has been shown to be specifically dependent upon Agr-regulated expression of alpha toxin [55,56]. We have shown that during invasive disease, this subversion allowed *S*. *aureus* to survive inside phagocytes [57] and speculate toxins may be functioning in a similar manner during colonisation, potentially facilitating the carriage status and the bacteria’s long-term survival and ongoing transmission.

## Surfactant-like toxins: Niche establishment and providing a competitive edge

A second class of toxins that attack host cell membranes are the surfactant-like phenol-soluble modulins (PSMs), which, to date, have been found to be expressed only by staphylococcal species. There are at least 8 genes identified that encode these short peptides (delta toxin, PSMα1, α2, α3, α4, β1, β2, β3, and PSM-*mec*), and their mode of action is to aggregate in the lipid bilayer of host cell membranes, leading to their disintegration [58]. As with the pore-forming toxins discussed above, we need to consider their potential role in the nose if we are to understand the selective benefit they confer to the bacteria, and again, a potential role in biofilm formation and survival inside phagocytes is a possibility (summarised in Fig 2). As with alpha toxin, PSMs have been shown to promote escape from the phagosome, which facilitates cytoplasmic replication and survival inside phagocytes [59]. With regard to biofilm, it has been shown that, during the later stages of *S*. *aureus* biofilm formation, PSMs are required for the development of the biofilm secondary structure. PSMs are thought to contribute to the formation of characteristic channels and macrocolonies, with PSM knockout mutants forming smoother, thicker biofilms lacking secondary structure. PSMs are involved also in biofilm detachment and dispersal, with PSM mutants showing reduced dispersal in murine models of catheter infection [60]. PSMs may therefore contribute to transmission to new sites of infection.

Another potential role for PSMs in nasal colonisation may be to enhance the ability of *S*. *aureus* to compete with other members of the nasal microflora. Individual bacterial species rarely exist in isolation but rather as multispecies populations, in which highly abundant members dominate, with many lower-abundance species co-occurring. Nutrient availability is a major driver of microbial competition and the battle for resources is fierce. The production of toxic compounds that supress and/or kill off competitors is a commonly deployed strategy used to competitively exclude sensitive, nonproducing isolates. The production of these secreted compounds enables the producer to kill off or inhibit its rivals, and there is some evidence that PSMs can act in such a role, in which PSMα1 and PSMα2 expressed by the notorious CA-MRSA lineage USA300 have been shown to exhibit considerable antimicrobial activity against *Streptococcus pyogenes* [61]. While still an offensive behaviour, it may be that the selective benefit these toxins confer is in the destruction of competing members of the nasal microbiome rather than host cells.

The lack of any motile capabilities may provide another benefit for the expression of PSMs by staphylococci. With no means of propulsion (flagella, pili, etc.), this genus of bacteria is entirely dependent upon external forces to move from one site to another, which is important, as overpopulation of a single niche would result in a rapid depletion of nutrients and potentially a triggering of an immune response. It is therefore interesting to note that, in vitro, the surfactant effect of PSMs on the environment surrounding the bacteria has been shown to contribute to the ability of *S*. *aureus* to move across agar surfaces in a process referred to as sliding [62]. Whilst this sliding activity may be solely an in vitro phenomenon, that the expression of the PSMs is highly density dependent provides a potential explanation for how this might assist in the early colonisation of the nasal cavity, as it could potentially facilitate the spreading of the bacteria across the lining of the nasal cavity once an optimal density at their initial attachment site has been reached.

## Protein synthesis–inhibiting toxins: Modulating the immune response

The inhibition of protein synthesis has catastrophic effects on host cells and is a pathogenic approach adopted by several bacteria. One of the most notorious examples of these is the shiga toxin expressed by *Shigella dysenteriae* as well as by recently emerged outbreak strains of enterohaemorrhagic *E*. *coli*. An interesting feature of this toxin is that it is encoded on a phage, so it is arguably a phage rather than a bacterial toxin and must therefore confer a selective advantage to both life-forms. The exogenous damage of cells lining the gut and the ensuing inflammatory processes provide a clear benefit to both phage and bacteria, as their transmission is effected by the production of diarrhoea. However, recent studies have highlighted immunomodulatory effects of this toxin that suggest that killing cells is not its sole effect. These toxins have been shown to up-regulate chemokine monocyte chemotactic protein-1 (MCP-1, CCL2) and IL-8 (CCL8) [63,64] and increase expression of cellular adhesion molecules ICAM-1, VCAM-1, and E-selectin on endothelial cells [65], suggesting that the recruitment of the cellular aspects of host immunity to the infection site is interfered with. The increased inflammation associated with immune cell recruitment would further exacerbate diarrhoeal symptoms, demonstrating that these toxins contribute to the transmission of the bacteria utilising processes beyond their offensive activity.

## Neurotoxins

There are other classes of bacterial toxins that, with our current understanding of their activity, make little sense from an evolutionary perspective. One such class is the botulinum and tetanus neurotoxins, which are zinc-dependent proteases that inhibit neurotransmission at neuromuscular synapses, resulting in either flaccid or spastic paralysis [66]. There is no evidence to suggest their expression directly confers an increased ability to colonise, replicate, or transmit beyond the infected host. However, if we consider this lethal activity alongside the ability of these bacteria to produce spores, then perhaps we can speculate about an indirect role in transmission. Sporulation provides a long-term survival strategy for the bacteria, allowing for transmission even after the host has been killed. Thus, perhaps by rapidly killing the host, the toxin decreases the chances of the host immune system clearing the infection, facilitating maximal spore formation and enhanced transmission. Alternatively, as a member of the gut flora of several animals, it is possible that they play an as of yet to be identified role in this niche.

## Superantigens

Superantigens are another class of bacterial toxins expressed in a wide range of bacterial genera (e.g., *Yersinia*, *Streptococcal*, and *Staphylococcal* species) that confer no apparent benefit to the bacteria, which raises the question of whether they exclusively function in the role of immune evasion. These toxins cross-link the class II major histocompatibility complex antigens on professional antigen-presenting cells to T-cell receptors, resulting in massive systemic release of pro-inflammatory cytokines, which can lead to fever, shock, and death of the patient. The induction of T-cell anergy is another feature of superantigens [67] and it’s tempting to speculate that at the trace levels expressed during colonisation, this subversion of the immune system might facilitate colonisation. However, a recent study found that the inactivation of a superantigen in 2 distinct lineages of *S*. *aureus* resulted in consistently higher bacterial loads in the nose when compared to their wild-type strain [68]. It is therefore clear that we do not understand the long-term benefit the expression of superantigens confer to their producing bacteria and should perhaps be grateful that, despite their ubiquitous nature, they rarely exert a pathogenic effect.

## Conclusion

In an attempt to understand the roles toxins play in the lifestyle of bacteria, we have adopted a perspective beyond their direct contribution to pathogenesis and allowed ourselves to speculate about alternative explanations for their prevalence. In doing so, we believe we have demonstrated how much we have yet to learn. This is particularly important when such virulence factors are being targeted during the development of novel therapeutics, in which interference with the expression or activity of those produced by bacteria causing an infection could have unforeseen consequences on other bacterial behaviours. It is perhaps a semantic problem that is blinkering us, relating to the term ‘toxin’, which we understandably take to mean as having a toxic effect. However, we believe it is critical to consider the potential of each toxin to be not only offensive but also defensive and perhaps contributing to a bacterial behaviour completely unrelated to pathogenicity, if we are ever to fully understand them and their producing microorganisms.
